# Toxocariasis Presenting as Encephalomyelitis

**DOI:** 10.1155/2011/503913

**Published:** 2011-05-05

**Authors:** Gregory Helsen, Stefaan J. Vandecasteele, Ludo J. Vanopdenbosch

**Affiliations:** ^1^Department of Neurology, Antwerp University Hospital, Wilrijkstraat 10, 2650 Edegem, Belgium; ^2^Department of Internal Medicine, General Hospital Sint-Jan, Ruddershove 10, 8000 Bruges, Belgium; ^3^Department of Neurology, General Hospital Sint-Jan, Ruddershove 10, 8000 Bruges, Belgium

## Abstract

We describe a farmer who presented with a clinical picture of a transverse thoracic myelitis. MRI showed inflammatory lesions in brain and thoracic spinal cord. Toxocariasis was suspected because of eosinophilia in blood and cerebrospinal fluid, and this diagnosis was confirmed immunologically. He was successfully treated with antihelminthics in combination with corticosteroids. Neurotoxocariasis is rare and diagnosis can be difficult because of the different and atypical clinical manifestations. It should be considered in every case of central neurological syndrome associated with eosinophilia.

## 1. Introduction

Toxocariasis is a parasitic zoonosis caused by larvae of *Toxocara canis* or *Toxocara cati*. These nematodes normally live in the small intestine of domestic dogs and cats, their definitive hosts. Most human infections are thought to be subclinical or self-limited, and clinical infections usually affect young children and manifest most commonly with pulmonary or hepatic disease. Clinical involvement of the central nervous system is rare and probably occurs as a result of hematogenous dissemination [1, 2].

## 2. Case Report

**Figure 1:**
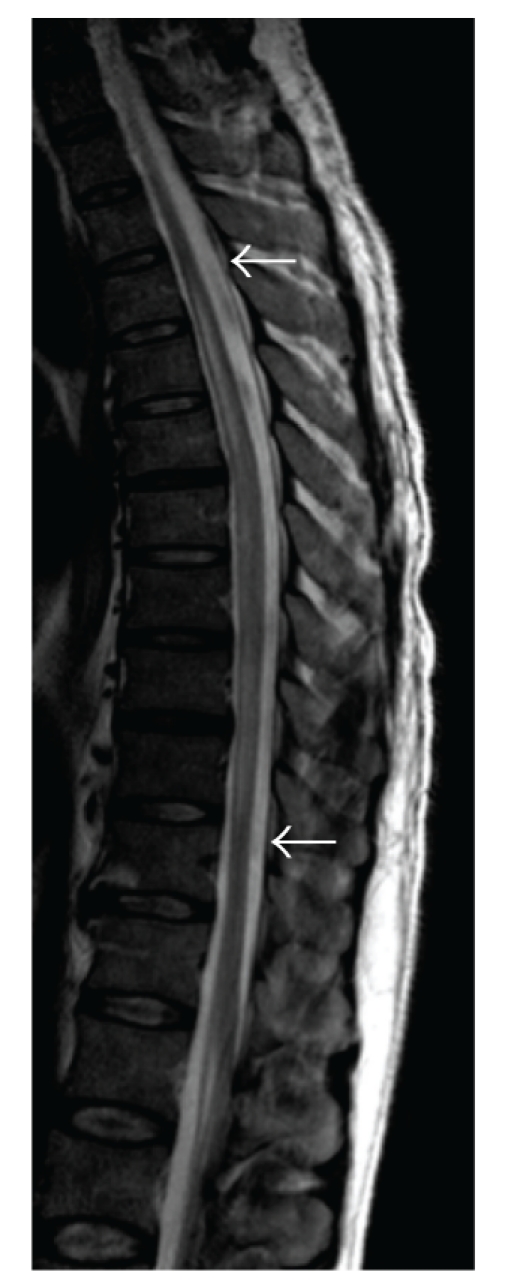
Sagittal T2-weighted spinal MRI shows an intramedullar hyperintense lesion extending from T4 to T10 (between the arrows).

**Figure 2:**
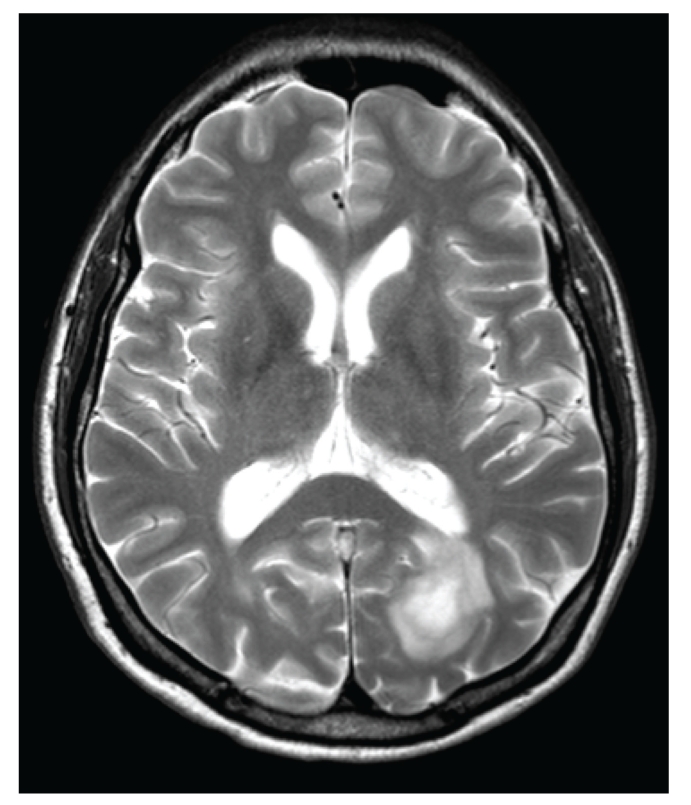
Axial T2-weighted brain MRI shows a large left-sided hyperintense occipital lesion.

**Figure 3:**
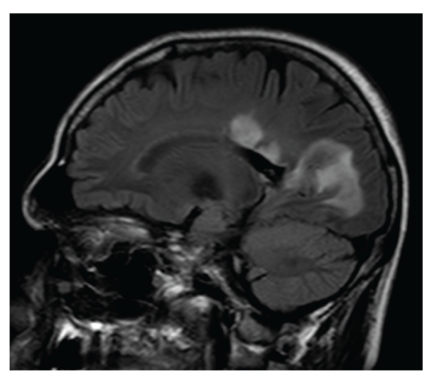
Sagittal FLAIR brain MRI shows a lesion cranial to the corpus callosum and the large occipital lesion.

**Figure 4:**
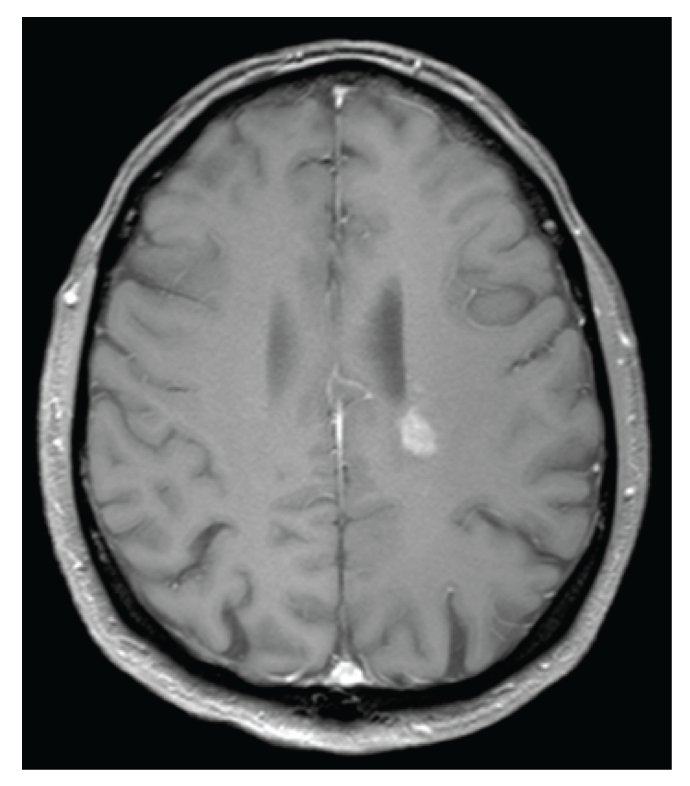
Axial postcontrast T1-weighted brain MRI shows a contrast-enhanced left-sided lesion in the periventricular white matter.

**Figure 5:**
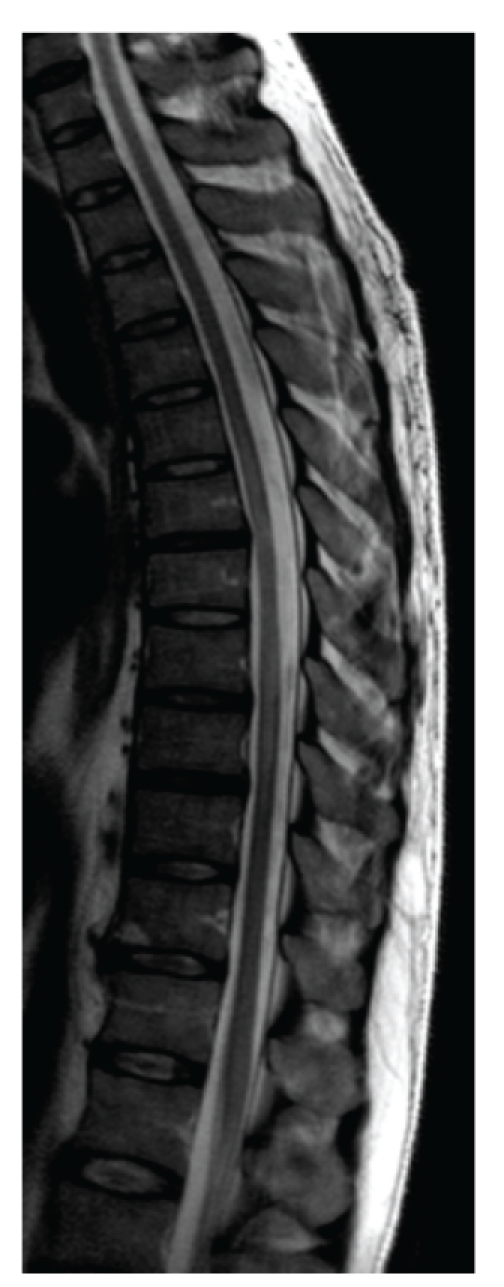
Sagittal T2-weighted spinal MRI 2 months later shows complete resolution of the lesion.

**Figure 6:**
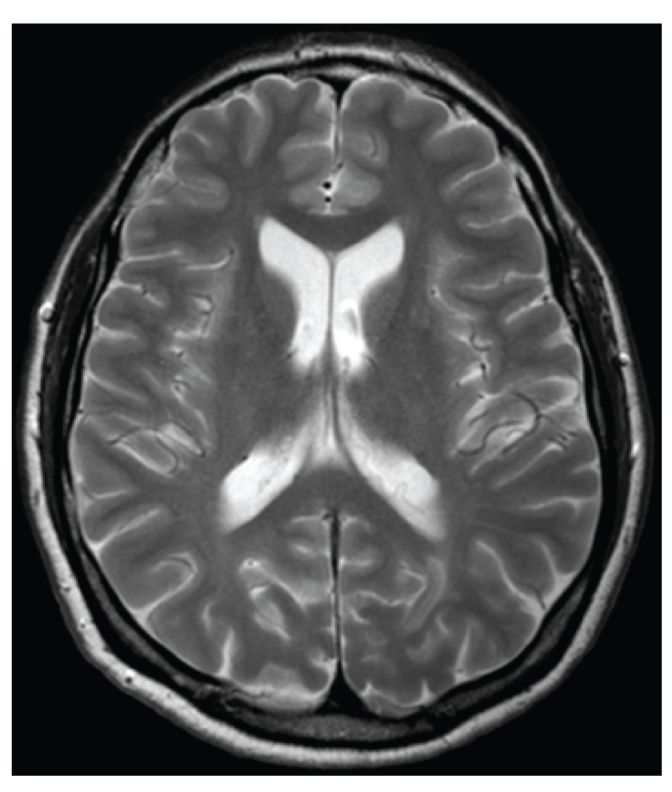
Axial T2-weighted brain MRI 2 months later shows complete resolution of the occipital lesion.

A previously healthy 45-year-old farmer presented at the emergency department with a tingling sensation and weakness in the lower limbs. The symptoms started 1 week earlier and were gradually progressive. He also noticed difficulty with micturition since the past few days. He did not have upper limb complaints nor back pain, and he denied any recent back trauma, infection, or vaccination. He had not been in foreign countries. On clinical examination, there was a symmetrical paresis of the lower limbs (global grade 4/5 on MRC scale, though the proximal muscle groups being slightly weaker), with a sensory loss of all modalities below thoracic dermatome level 10, brisk deep tendon reflexes in the lower limbs, and bilateral Babinski signs. Over the next 2 days, his case deteriorated into paraplegia with anaesthesia in the lower limbs and total loss of control of bladder and bowel. Spinal MRI showed an intramedullary T2-weighted hyperintensity extending from thoracic level 4 to 10 without contrast enhancement (Figure 1). Brain MRI showed multiple T2-weighted hyperintense lesions, the largest lying posterior to the left lateral ventricle (Figures 2 and 3). The supratentorial lesions showed contrast enhancement (Figure 4), the one in the pons did not. Examination of cerebrospinal fluid (CSF) revealed 25 leukocytes/*μ*L with 87% lymphocytes, 9% eosinophils, 33 mg/dL of protein, and 62 mg/dL of glucose; IgG index was normal (0.62); there were no oligoclonal IgG bands. The relative amount of peripheral blood eosinophils was increased to 21% with the total white blood cell count being normal (8.300/mm^3^). C-reactive protein and routine biochemical blood tests were all normal. Ophthalmologic examination was normal, as were thoracic and abdominal CT scans. Treatment was started with intravenous methylprednisolone (1 g q24h for 5 days). The presence of eosinophils in the blood and CSF suggested a parasitic infection, and therefore oral mebendazole (200 mg q12h) was added. Antibody titer against *Toxocara canis* using ELISA (for excretory-secretory antigen) was 1 : 500 positive in blood and 1 : 32 positive in cerebrospinal fluid. A broad screening for other neurotropic infections (including *Taenia*) was negative. The patient did not own domestic dogs, but multiple cats were free-living on his farm. He regularly soiled his hands while feeding hay to his cattle, and he did never wash his hands before meals. A diagnosis of *Toxocara* encephalomyelitis was made, and he was further treated with oral albendazole 400 mg q12h (11 mg/kg body weight) for 16 days and oral methylprednisolone starting with 64 mg q24h and slowly tapering off the dosage. He slowly recovered. About 2 months after the onset of symptoms, he could walk independently but still with slight disability. Imaging of spinal cord and brain at that time showed complete resolution of lesions (Figures 5 and 6).

## 3. Discussion

Human toxocariasis is a zoonosis caused by infective larvae of *Toxocara canis* or (less often) *Toxocara cati*. These nematodes live as adult worms in the intestinal tract of dogs and cats, respectively. Humans are usually infected by ingestion of embryonated eggs in soil, most often on contaminated hands [1, 2]. The migration of larvae to various tissues causes pathologic and immunologic responses with granulomatous inflammation and eosinophilia [2–4]. Visceral involvement of the disease is called visceral larva migrans (VLM) [2]. Most *Toxocara* infections are probably subclinical or benign and self-limiting. The typical patients are children between the ages of 2 and 7 years with a history of geophagia and exposure to puppies or kittens at home or in public parks [1–3]. Some experts have disputed the role of dogs as transmitter because up to half of patients do not own a pet and cannot recall any close animal contact [4]. The clinical signs of VLM are usually associated with hepatic and pulmonary larval migration and include abdominal pain, decreased appetite, restlessness, fever, coughing, wheezing, asthma, and hepatomegaly. Infection is usually characterized by marked and chronic eosinophilia, leukocytosis, and hypergammaglobulinemia [1–3]. In ocular larva migrans (OLM), involvement is restricted to the eye and the optic nerve. OLM typically presents as unilateral vision impairment that is sometimes accompanied by strabismus. The most serious consequence of infection is invasion of the retina, leading to granuloma formation. Blindness is a common complication [2].

Neurological manifestations of toxocariasis are rare. In the English language medical literature from 1950 to 2007, we found only 31 cases of neurological toxocariasis in humans [1, 5, 6]. Possible clinical syndromes are encephalopathy, meningoencephalitis, transverse myelitis, psychiatric disturbances, focal or generalized seizures, epilepsy, and death [3]. For a brief overview of the main clinical and paraclinical data of 21 patients from 1956 until 2002, we refer to the article by Moreira-Silva et al. [7]. In literature, we have encountered only 5 previous cases of *Toxocara* myelitis (with MRI and positive serology in blood and CSF), all of them made a good recovery [5, 6]. Recently, a 2-year-old child with *Toxocara* encephalomyelitis has been reported, pointing out the possible resemblance to acute disseminated encephalomyelitis [6].

The enzyme-linked immunosorbent assay (ELISA), that uses antigens secreted by the second-stage larvae, is the best indirect test for diagnosing infection. The ELISA has a reasonably high sensitivity of approximately 78% at a titer greater than 1 : 32 [2]. A disadvantage of this test is that anti-*Toxocara* antibodies measured by ELISA were found to persist for up to 2.8 years in infected adults so that the presence of these antibodies alone does not distinguish between current and past infections [1]. In northern industrialized countries, the seroprevalence of this infection is 5% in urban adults and up to 40% in children and rural farmers. In the West Indies and Bali, seroprevalence rates approach 80% [1, 4]. Therefore, one should also take into account blood eosinophilia, which is considered to be a reliable indicator of active helminthiasis, serum IgE, and nonspecific tests such as erythrocyte sedimentation rate and C-reactive protein in case of VLM. Neurological syndromes with *Toxocara* infection of the central nervous system are generally nonspecific, and peripheral eosinophilia is often lacking. MRI can detect granulomas located cortically and subcortically, and these may appear as hyperintense foci on T2-weighted and proton density images. When associated with eosinophilia in the CSF, such images suggest a *Toxocara* infection. The presence of *Toxocara* larvae in cerebrospinal fluid, in brain tissue or in the meninges, and/or a positive anti-*Toxocara* antibody titre in CSF, can confirm the diagnosis [1]. In cases of cerebral and spinal parenchymal lesions in combination with eosinophilia in blood and CSF, one should also consider neurocysticercosis, especially in endemic areas. Neurocysticercosis typically presents with epileptic seizures and one or multiple cystic brain lesions. Although very rare, cases of extramedullary and intramedullary spinal neurocysticercosis have been described [8, 9]. 

Acute VLM should be treated. Different treatment regimens exist. Diethylcarbamazine (DEC), if available, is often considered the most effective treatment [1, 4]. It is given in a dosage of 3 to 4 mg/kg body weight in 3 divided doses daily for 21 days. DEC should not be given together with corticosteriods because of being antagonism of corticosteriods, which partially inhibit DEC's mechanism of action. Therefore, DEC and corticosteriods must be given sequentially. The drug has been associated with a high rate (28%) of neurological side effects (especially dizziness and headache) and (in 10%) a Mazzotti-like reaction (itching, urticaria, and/or oedema). Mebendazole (MBZ) is available in many countries and would appear to be a good alternative to DEC, for example, if the occurrence of major DEC-related side effects is feared. Dosage is 20 to 25 mg/kg body weight (in a single dose) daily for 3 to 4 weeks. The drug is practically insoluble in water and should be taken with a fatty meal [1, 4]. Albendazole (ABZ) is given at 10 to 15 mg/kg body weight in 2 divided doses daily for 5 to 15 days and is considered by several authors as the treatment of choice [2, 3]. Thiabendazole (TBZ) is not recommended because of a high rate of side effects (including cholestasis and hepatitis) [1]. Corticosteroids are sometimes added to antihelminthics to reduce the acute inflammatory and immunologic manifestations [1–3]. In neurotoxocariasis in particular, corticosteroids are thought to be important because the central nervous system lesions are more likely to represent inflammatory reactions rather than abscess formation [1, 5].

## 4. Conclusion

We present this case because of the unusual combination of spinal cord and cerebral lesions in toxocariasis. To our knowledge, our patient represents the first adult described in English literature with concomitant involvement of brain and spinal cord (documented with MRI). Neurotoxocariasis can result in varying and atypical neurological manifestations, including one that simulates acute disseminated encephalomyelitis. One should consider neurotoxocariasis in every central neurological syndrome associated with eosinophilia. Treatment consists of the combination of antihelminthics and corticosteroids. A detailed history taking is important, especially with regard to travelling. *Toxocara canis* occurs worldwide. If the patient has recently travelled abroad, differential diagnosis of eosinophilic meningitis/myelitis is more extensive and includes *Taenia solium*, *Gnathostoma spinigerum*, *Angiostrongylus cantonensis, and Baylisascaris procyonis *[8–10].
